# Supplementary figures and images for: Limited evidence for executive function load impairing selective copying in a win-stay lose-shift task
Source: PLoS One. 2021 Mar 4;16(3):e0247183. doi: 10.1371/journal.pone.0247183 (PMC7932141; doi:10.1371/journal.pone.0247183)

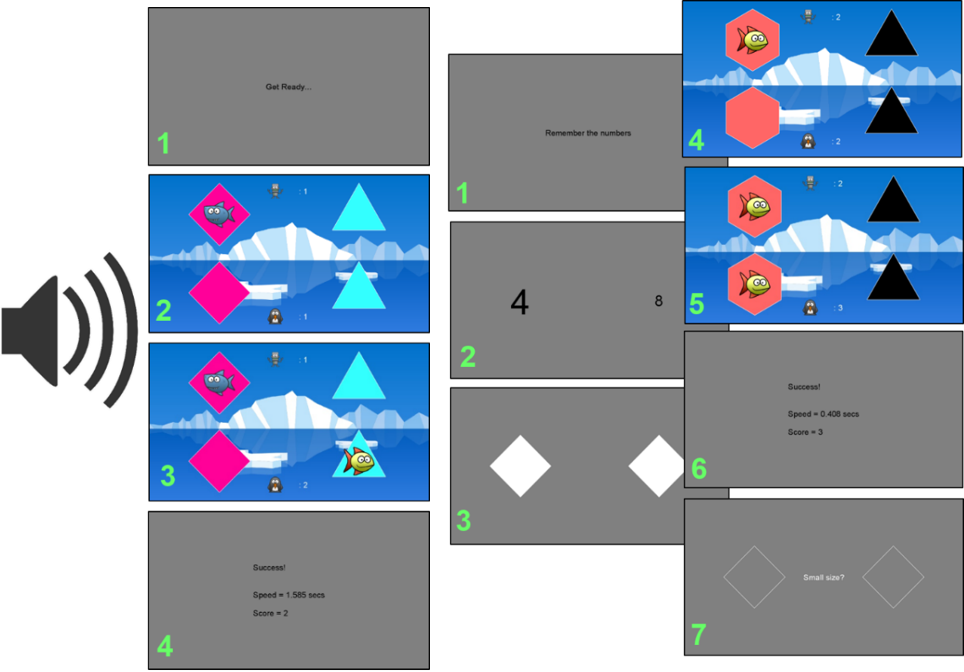

Supplement: S1 Fig — (TIF) [file pone.0247183.s001.tif]
