# Supplementary material for: Limited evidence for executive function load impairing selective copying in a win-stay lose-shift task
Source: PLoS One. 2021 Mar 4;16(3):e0247183. doi: 10.1371/journal.pone.0247183 (PMC7932141; doi:10.1371/journal.pone.0247183)
Supplement: S1 File — (DOCX) [file pone.0247183.s017.docx]

# Limited evidence for executive function load impairing selective copying in a win-stay lose-shift task: Supplementary information

### Detailed methods: Binary Choice Task, Pilot, E1 & E2

Code to run all of the tasks for this can be found here: <https://osf.io/bfg3x/>

Participants played as a penguin avatar, and the task required participants to find desired hidden objects (fish) behind coloured shapes presented on screen, and to avoid undesired objects (sharks). Participants played against the computer (represented as a robot avatar) to find as many fish as possible.

Participants were shown an information trial in which the computer selected a subset of the shapes presented on screen and displayed the hidden object(s). The participant was then presented with the same shapes again and was required to make their own selections with the goal of finding the desired object(s). The information trial was either successful (fish were found) or unsuccessful (sharks were found). Reward location was fixed, so to perform optimally participants needed to employ a win-stay, lose-shift (WSLS) strategy.

For pilot (P) & E1 trials the task was a binary choice, with the computer and participant making one selection from two shapes. In E2 two selections were required from four shapes. On each trial, after a 2s ready screen participants were shown an information trial in which the computer selected one (P & E1) or two (E2) of the shape stimuli on the screen to reveal the object(s) underneath. The colour and shape of stimuli were pseudo-randomly determined. The number of sides was randomly selected from a choice of 3-6 (P & E1; for E2 the choice also included a circle), with the caveat that on each trial no shapes could have the same number of sides. The colour of the shapes was randomly selected from a pre-specified list of 16 colours chosen from opposite sides of a colour chart (see figure S1), with the caveat that on each trial no shapes could be the same colour. Shape colours were selected from a limited list rather than randomly generated to prevent colours that were technically different but functionally indistinguishable from being selected. Colours were also intentionally chosen to be salient against the task background (see figure 1, main text).


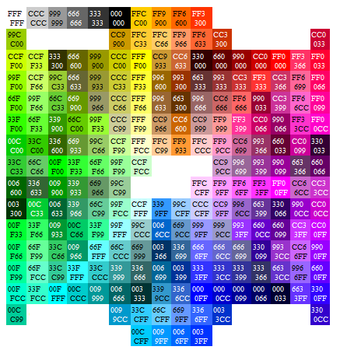


Figure S1: Example colour chart used to select colours that were as visually different to each other as possible

The information trial was either fully successful (a fish was found; two fish in E2) or fully unsuccessful (a shark was found; 2 sharks in E2). In E2 there was also the option for a trial to be partially successful (one fish and one shark were found). For the selective copying conditions, in P & E1 there was a 50% chance of showing a successful or unsuccessful information trial, and whether the computer selected the shape on the left or the right of the screen with an equal balance of all possible success and side options. In E2 there was an equal balance between fully successful, fully unsuccessful and partially successful trials with an equal balance of all possible stimulus pairings. There was therefore an equal balance of successful and unsuccessful selections on each side of the screen. These were presented in a fully random order. In the always copy and never copy conditions all trials were fully successful or fully unsuccessful.

The information trial was displayed by either a fish or a shark appearing in the centre of the shape stimuli after a random delay of between 800-2400ms (in line with previous reaction time literature:, (e.g Cook & Bird (2011)). After each information trial the visual counter of the computer’s score next to its avatar would increase by the number of fish revealed in that trial (0-2). When the task was not intended to impose a memory load (Pilot, E1, and some conditions of E2) all shapes, the object(s) found and their location would remain visible to the participant for the duration of the following test trial. When the task was intended to impose a memory load (some conditions of E2) the objects found during the information trial would disappear as the test stimuli appeared.

Participants then completed a test trial by making their own selection from the same shape stimuli which were presented again directly underneath the information stimuli. In P & E1 the test stimuli appeared as soon as the information trial selection had been displayed. In E2 the test stimuli appeared after a random delay between 800-2400ms, to allow an equal delay when the memory load was present or absent. The participants’ aim was to find all the fish as quickly as possible. Responses were collected using a Black Box toolkit 4 button response pad In P and E1 participants were instructed to only use the two outermost buttons, with the left button corresponding to the left stimulus and the right button corresponding to the right stimulus. In E2 the buttons left to right corresponded to the stimuli left to right. After selecting a shape, either a fish or a shark was displayed in the centre of that shape stimulus. If successful, the visual counter of the participant’s score next to the penguin avatar would increase by one. In E2 a second selection would then need to be made. On partially successful trials the reward structure was fixed so that if employing a correct WSLS strategy there was a 50% chance of finding a fish or a shark in either of two available locations. This screen was displayed for a further 1s, and then participants were given immediate feedback about their performance on a screen that told them if they had been successful, and if so told them their reaction time (in seconds) and a running total of their score so far. If unsuccessful they were again given immediate feedback and a reminder that they were looking for fish and aiming to avoid sharks, but were not given their reaction time or a running total of their score. This screen was displayed for 2s and then either the *working memory* test trial (in P, *working memory* condition) or a new trial of the choice task would commence. In E2 a 3s time limit was imposed on the first response. If a selection was not made within 3s the trial would end and a message would display on screen reading ‘trial missed, response must be given within 3 seconds’ and one point would be removed from the total.

### Detailed Methods: Dual Tasks (Pilot)

Dual Task 1: *Incongruence*

*EF task:* Participants listened to a series of auditory tones, in sets of either 1 or 2 tones, through headphones and were instructed to respond to each set of tones with the opposite number (i.e. either 2 or 1) of mouse clicks. Tone sets were played every 3 seconds. There was a 50% chance of a single tone and a 50% chance of a double tone, presented in a fully random order.

*Control task:* Identical to the EF task, but participants were instructed to respond with the same number of clicks as tones.

This is similar to the executive function distractor task used by Qureshi, Apperly and Samson (2010).

Dual Task 2: *Inhibition (withholding*

*EF task:* Participants listened to a series of auditory tones, in sets of either 1, 2 or 3 tones, through headphones and were instructed to respond to each tone with the same number of mouse clicks as tones, unless the number of tones matched a pre-specified number for which they were instructed to withhold their response. Whether this pre-specified number was 1, 2 or 3 was randomly determined at the start of the testing session. Tone sets were played every 3 seconds. The number of tones played was split at 40%, 40%, 20% with the number that should have responses withheld presented less often.

*Control task:* Identical to the EF task, but participants were instructed to respond to every tone with the same number of mouse clicks as tones, with no numbers requiring withholding a response.

This is similar to the ‘inhibition’ executive function distractor task used by Bull, Phillips and Conway (2008).

Dual Task 3: *Switching*

*EF task:* Participants listened to a series of auditory tones, in sets of either 1 or 2 tones, through headphones and were instructed to respond to each tone with the same number of mouse clicks as tones. After an auditory cue they were required to switch to responding by clicking once more than the number of tones heard. Participants were instructed to switch between clicking the same number of times as the tones heard and clicking once more than the tones heard each time they heard the auditory switch cue. Tone sets were played every 3 seconds. There was a 50% chance of a single tone and a 50% chance of a double tone, presented in a fully random order. Switch cues were played at an average rate of 1 cue for every 5 sounds played, presented in a pseudo random order which meant the series of tones could not begin with a switch cue, and a switch cue could not be played immediately after another switch cue.

*Control task*: Identical to the EF task, but participants were instructed to continue to respond to each tone with the same number of mouse clicks as tones heard for the duration of the task and no switch cues were played.

This is similar to the ‘switching’ executive function distractor task used by Bull, Phillips and Conway (2008).

Task 4: *Updating*

*EF task:* Participants listened to a series of auditory tones, in sets of either 1 or 2 tones, through headphones. They were instructed to respond by clicking the mouse once more than the number of tones heard in the *previous* set. Participants were instructed not to click in response to the first set of tones, as there was no previous tone set to respond to. For example, if the tone sequence 2, 1, 2… was heard the participant should respond with the click sequence 0, 3, 2, 3…. Tone sets were played every 3 seconds. There was a 50% chance of a single tone and a 50% chance of a double tone, presented in a fully random order.

*Control task:* Identical to the EF task, but participants were instructed to respond by clicking once more than the number of tones heard in the *current* set.

This is similar to the ‘updating’ executive function distractor task used by Bull, Phillips and Conway (2008).

Task 5: *Working Memory*

*EF Task:* This dual task was presented between trials of the main task. At the start of each trial participants were given a visual instruction of “remember the numbers”. They were then presented with two numbers on screen, one of which was clearly much larger in font size than the other, and one of which was larger in numerical value than the other. On 50% of trials the large value number was in larger font, and on the remaining 50% the large value number was smaller in font size. These trials were presented in a fully random order. The numbers were pseudo-randomly selected from the digits 2-9, with the constraint that the numbers could not be the same. The numbers were displayed for 3s and then masked with a white square for 0.5s. After each trial of the main task participants were presented with a screen with 2 unfilled white squares and a question in the middle. This question would ask which number was larger/smaller in *size* or which number was larger/smaller in *value* and the participant was instructed to select either the left or the right square to relate to the numbers presented at the beginning of the trial. The choice of question was randomly picked from a possible 4. There was no time limit for responses to this question. After making a response participants were given immediate feedback about whether they had been correct.

*Control task:* The format of the task was the same. However, instead of being shown numbers to remember at the beginning of the task participants were instructed to look at 2 fixation crosses, placed in the same screen position as the numbers in the *EF Task.* After each trial of the main task participants were then given a very simple numeracy task and had to say which of 2 numbers presented on the screen was larger or smaller in value. These numbers were randomly picked from a list of large and small numbers. The question was a random choice of the 2 questions (larger or smaller).

This is modelled on the executive function distractor task used by Coutinho, Redford, Church, Zakrzewski, Couchman and Smith (2015).

### Analysis of dual task data: E1

Participants all completed a different number of trials of the dual task, as their total trial number depended on the speed at which they completed the main task. Analysis of the dual task performance was therefore capped at 124 trials, as this was the minimum number of trials completed by all participants in either block (range EF block: 124-147; range control block: 153-169).

Participant accuracy was at ceiling in the control condition, and significantly above chance in the executive function condition: performance in each block of each condition was significantly above a chance level of 50% as shown by binomial testing (p<.001 for all conditions).

Table S1: Mean accuracy in each strategy group and block condition

| **Strategy Group Membership** | **Block Condition** | **Mean Accuracy** |
| --- | --- | --- |
| **WSLS** | EF Dual | 78.0% |
|  | Control Dual | 97.1% |
| **LS** | EF Dual | 82% |
|  | Control Dual | 97.2% |
| **WS** | EF Dual | 73.6% |
|  | Control Dual | 96.7% |

Success on each trial of the dual task was analysed using a binomial linear mixed effects model with fixed effects of group, block condition, scaled item number and their interactions, and participant ID as a random effect. This model was significantly better than the null model (χ2(11)=4881, p<.001). Accuracy was significantly lower in the EF block (b=-2.38, SE=0.095, z=-25.1, p<.001) and accuracy got lower as trial number increased, in the EF block only (b=-2.53, SE=0.228, z=-11.1, p<.001; see SI figure S3). There was a significant interaction between group, block condition and item number (b=1.01, SE=0.325, z=3.10, p=.002). Post-hoc analysis using the emtrends function in R showed that accuracy declined *over item number* significantly less in every strategy group in the control block compared to every strategy in the EF block (p<.001 for all comparisons). In the EF block the WSLS group showed significantly more decline over item number than the WS and LS groups (p=.001 and p=.009 respectively) but there was no difference between the LS and WS groups (p=.999). However, a post-hoc Bonferroni-Holm correction of all pairwise comparisons of group membership indicated there was no significant difference between overall accuracy between strategy groups (p>.99 for all comparisons), indicating that the EF impact on the different groups was indeed similar. There was either no offloading of the dual-task impact onto the audio task rather than the search task, or the amount of offloading was the same across strategy groups.


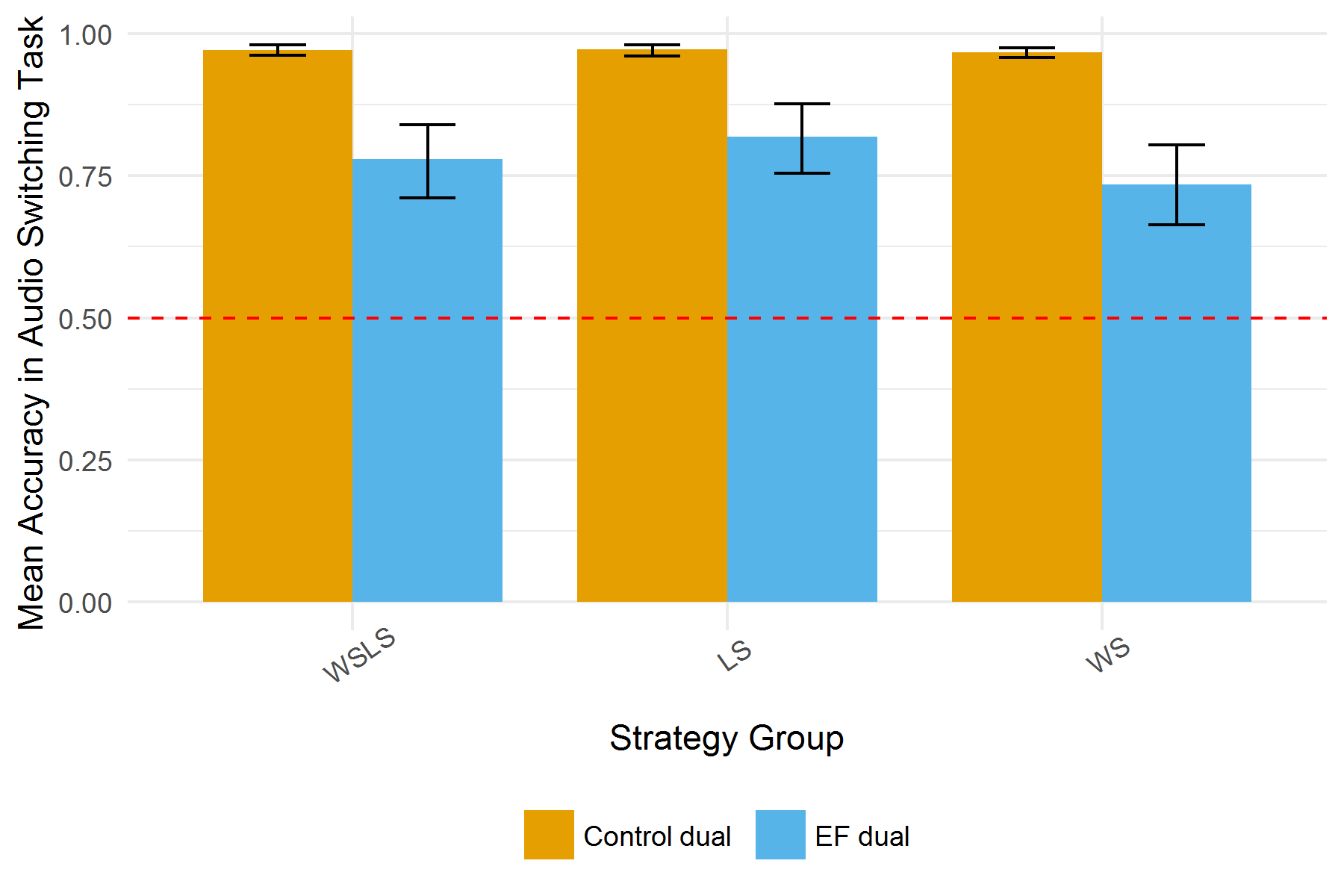
Participant accuracy was at ceiling levels in the control block and significantly above chance for the EF block in all conditions (p<.001 for all conditions) – see figure S2.

Figure S2: Mean accuracy in switching task per condition. Error bars represent bootstrapped 95% confidence intervals. Red line indicates chance performance.

The interaction between block condition and item number is shown in figure S3. The decline in accuracy in the EF block is expected to be due to the fact that accidentally missing or not hearing a single switching cue could mean a participant believed they were responding accurately, but was actually wrong on all trials after that point, unless another cue was missed.

| 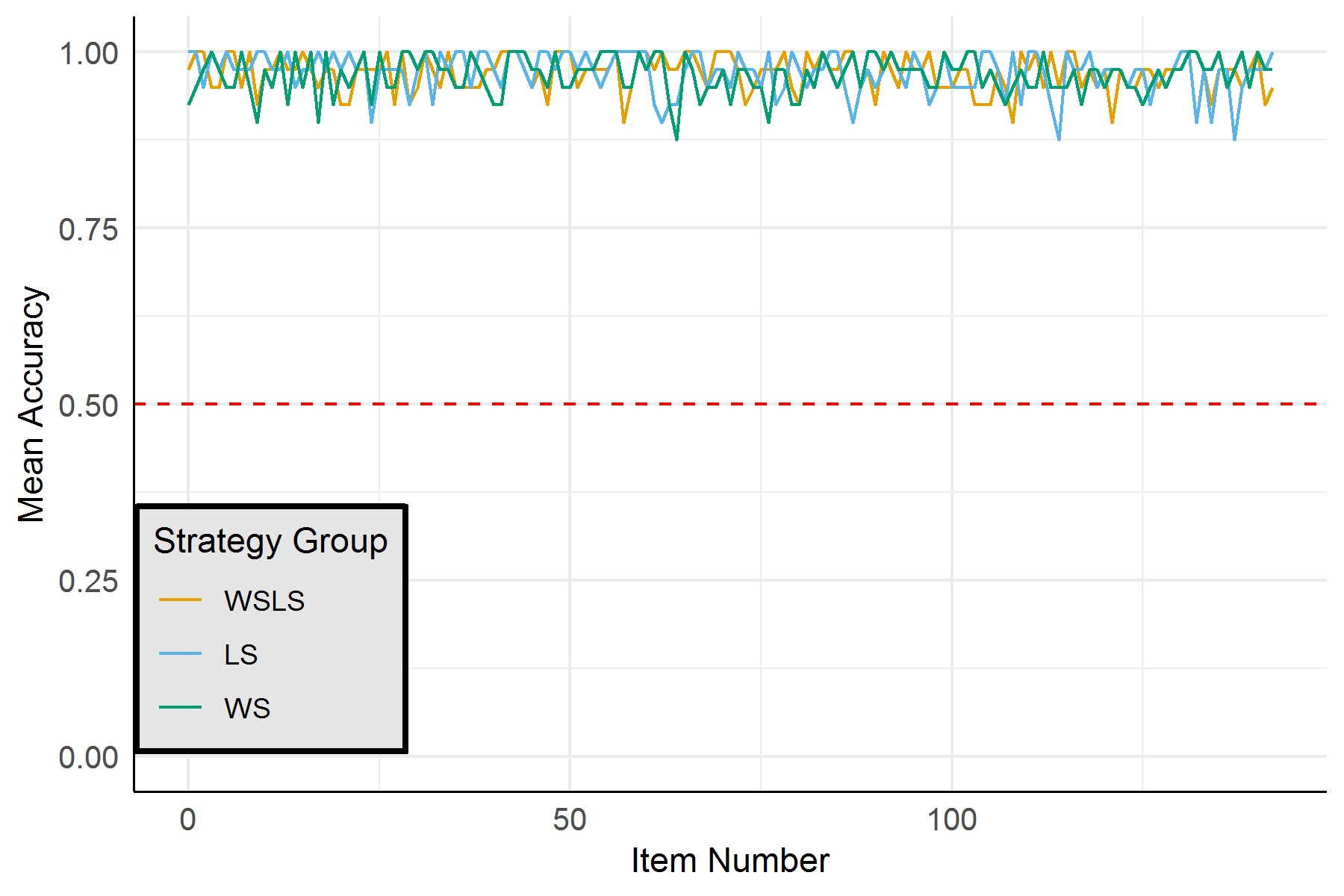 |  | 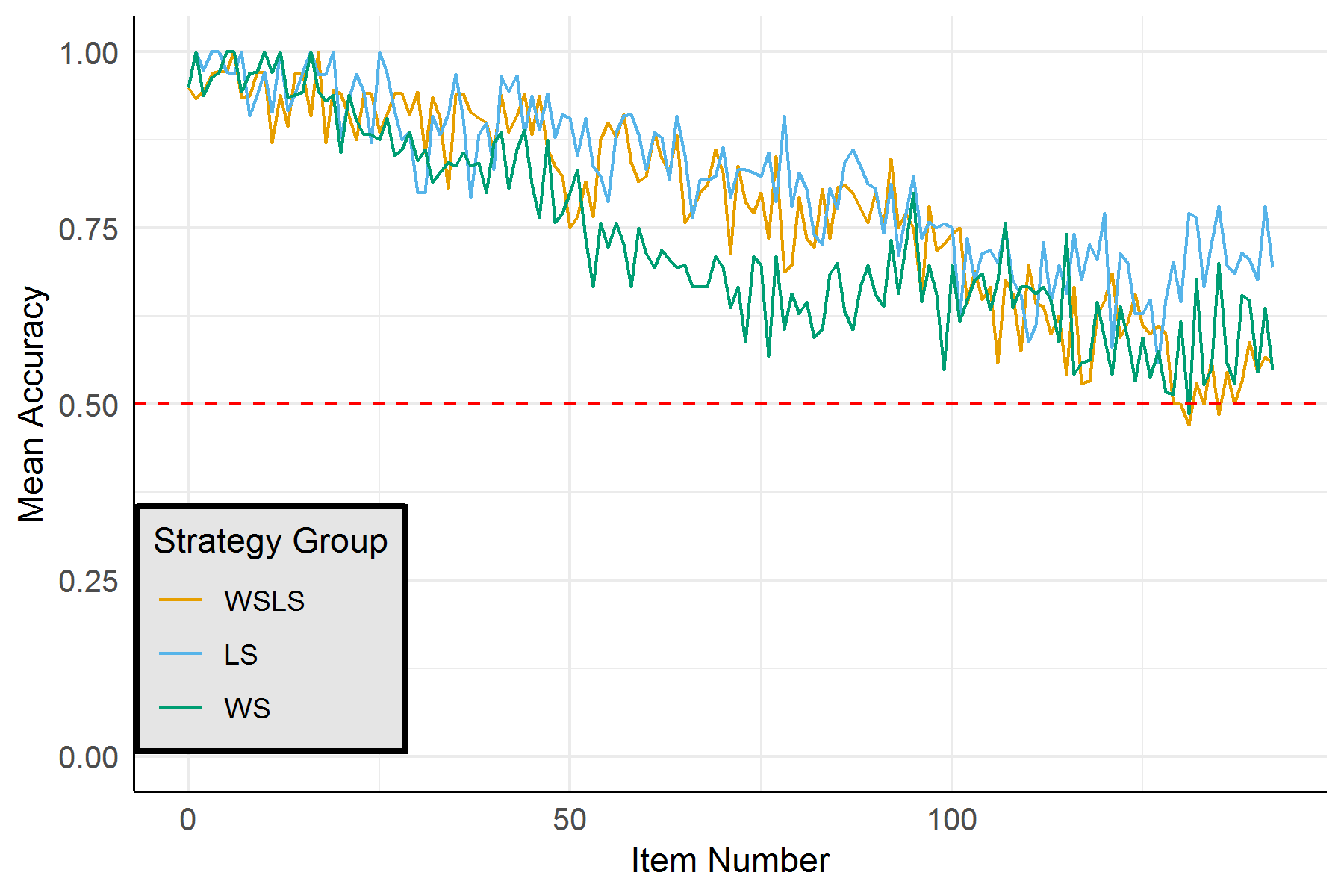 |
| --- | --- | --- |
| Figure S3: Decrease in mean accuracy in dual task in executive function block (right panel) compared to consistent high accuracy in the control block (left panel). Dashed line indicates chance performance. | | |

### Detailed Methods: Metacognition Task (E2)

Participants were shown two circular patches of white dots on a grey background, with the question ‘Which circle has fewer dots?’ written beneath it. To generate the dot circles one circle was randomly generated with a density of between 500 and 800 dots. A second circle was generated by then adding or removing a fixed percentage of the dot density, based on the difficulty level. There were 7 difficulty levels, each twice as difficult as the level before. Level 1 had a difference of 64% between the patch densities, so if one patch contained 500 dots the other would contain either 820 or 180 dots. The difference between the patches reduced by half at each difficulty level until level 7 which had a difference of 1% between the patches, so if one patch contained 500 dots the other would contain either 505 or 495 dots. The top end of the difficulty scale was designed to be functionally impossible to ensure participants would be guessing on a certain number of trials, to encourage them to use the full scale of confidence. Overall, the difficulty levels were set through pilot testing, to try to achieve accuracy levels of around 70% in the task.

There was an even balance of all 7 difficulty levels in each round, presented in a fully random order. The reward structure was fixed so that there was a 50% chance of the target patch (the patch with the lower density) being on the left of the screen, so the target should have appeared on the left or right side of the screen an equal number of times.

Participants were required to select one of two buttons on the button box to choose the patch on the left or the right of the screen. They needed to make their selection within 5 seconds, otherwise the trial would end and a message would display on screen reading ‘Trial missed, response must be given within 5 seconds’ and one point would be removed from the total. If the trial was missed an opportunity would not be given to report a confidence rating.

Once a selection had been made participants were asked to rate their confidence from 1-4 using one of the four buttons on the button box, with each button representing a number. After a response had been given a red ‘x’ would appear on the scale at the confidence level selected for 1s, and then a score screen would appear showing how many points had been gained or lost. Points were gained for correct patch discriminations and lost for incorrect patch discriminations, and the number of points gained or lost corresponded to the confidence level given. For negative scores the score screen would show in red text, and for positive scores it would show in white. During trials a running total score would be shown in the top right corner of the screen. This score could be negative, and negative total scores were displayed in red.

### Detailed Methods: Changes to Audio Switching Task for E2

In E2 the audio switching task was made slightly easier by removing the requirement of the participant to remember whether they should be clicking the same number of times as tones played, or once more than the number of tones. The switching task now had two different switch cues, one to indicate that participants should start adding one to the tones they heard and one to indicate that they should click the same number of times as tones they heard. These different cues were noticeably distinct, and logically set to ensure participants should remember which cue was which: the cue to start adding one to tones consisted of 2 short buzzes and the cue to stop adding one was a single buzz. Participants were informed about the meaning of these cues during the instructions of the task and played an example of both cues as well as given a small number of practise trials using them before the training round.

### Accuracy Threshold Settings: Experiment 2

For both the metacognition and WSLS search tasks a performance threshold of 75% was set as a requirement for passing the initial training round. For the WSLS search task this was intended to be quite generous as previous testing had shown ceiling levels of accuracy in this task. The accuracy threshold was only intended to act as a motivator for participants to give the task sufficient attention to ensure they could continue on to the full testing rounds of the experiment.

A score threshold of 75% in the metacognition task allowed for an accuracy of around 65% in the perceptual task, slightly lower than that found in initial pilot testing of the task, and then to perform at around chance at the confidence judging stage. This was to ensure participants with naturally lower levels of metacognition were not screened out, while ensuring participants who were performing at chance in the visual perception task, due to lack of concentration or poor visual acuity (and therefore whose metacognitive judgments were either not informative or always at the lowest level of confidence) were.

Difficulty level in the visual perception task had been set through piloting at a level that meant participants were expected to score on average around 70% accuracy.

For the audio dual task the performance threshold was set at 65% accuracy. This needed to be reached in both the executive function and control rounds. This was to ensure participants were performing significantly above chance in both tasks (based on a predicted 80 – 85 trials completed in each condition of the training rounds). Chance level was presumed to be 50% accuracy for both EF and Control rounds as participants should always have been choosing from one of two response options: click once or twice on non-switching trials, and click twice or three times on switching trials. This accuracy threshold assumed participants had a clear understanding of the task instructions and were attempting to respond appropriately, and was essential to include to ensure data collected were accurately reflecting behaviour under dual task conditions. If not attempting, or not understanding how, to respond in an accurate manner participants would not be considered to be taking part under genuinely dual task conditions and data from those participants would therefore be misleading and inaccurate.

Participants were required to pass all three tasks in order to progress to the full testing rounds.

### Exclusions from the full data set: Experiment 2

57 participants completed the training phase of the experiment but did not complete the full testing phase:

- 6 were excluded due to experimenter or technical error during testing
- 1 was excluded as a fire alarm sounded during the second block of testing and the building needed to be evacuated
- 1 was taken unwell during the second testing break and left voluntarily.
- The remaining 49 participants were excluded as they did not pass their initial training round:
  - 3 participants did not fully understand the instructions, due to language barriers which were only made known to the experimenter after the task ended. This meant they were often responding unintentionally incorrectly to trials, despite giving full attention and effort to the task.
  - 6 participants failed on the basis of their accuracy scores on the metacognition task.
  - 2 participants failed as they did not respond to any of the audio tones and did not respond to prompts from the experimenter to remind them of the task instructions.
  - 1 participant failed based on their WSLS task score.
  - The remaining 37 participants failed based on their audio-task accuracy. Audio task accuracy from participants who failed the training round was generally well below the required level to pass with a mean only slightly above chance performance (see supplementary figure S4).
- Participants who did pass the training round performed well, with accuracy levels close to ceiling (see figure S4).

| 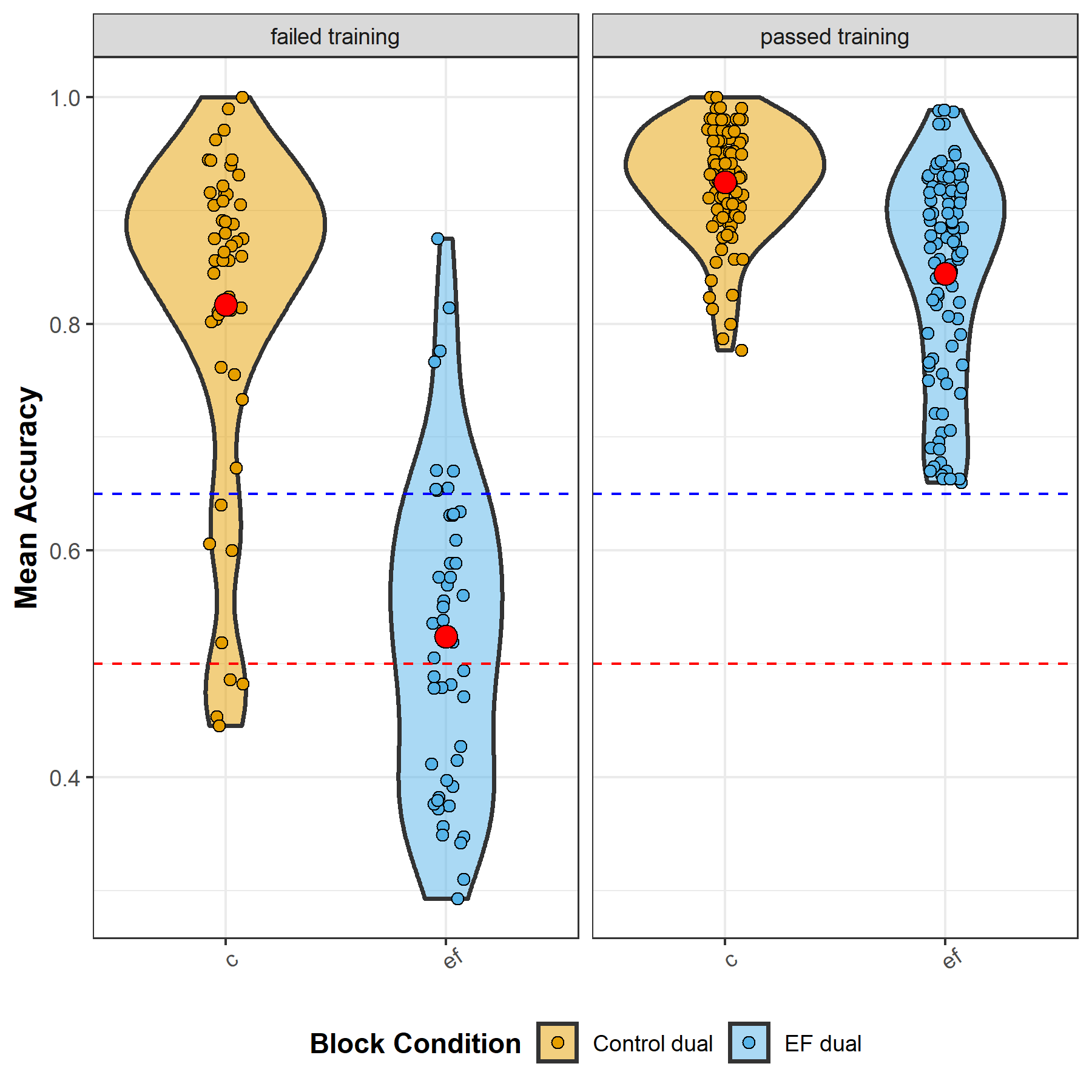 |
| --- |
| Figure S4: Accuracy in the training round of the audio dual task for participants who passed and failed training. Red dots indicate mean of each block. Red dashed line indicates chance performance, blue dashed line indicates passing threshold. Some participants appear to be above the passing threshold but still appear in the ‘failed training’ section as they may have passed the accuracy threshold in one block, but a pass in all blocks & conditions was required to continue to full testing. |

### Interactions in E2

The below figures and analysis give an additional representation of some of the interactions from E2.

Group, block condition and memory load:


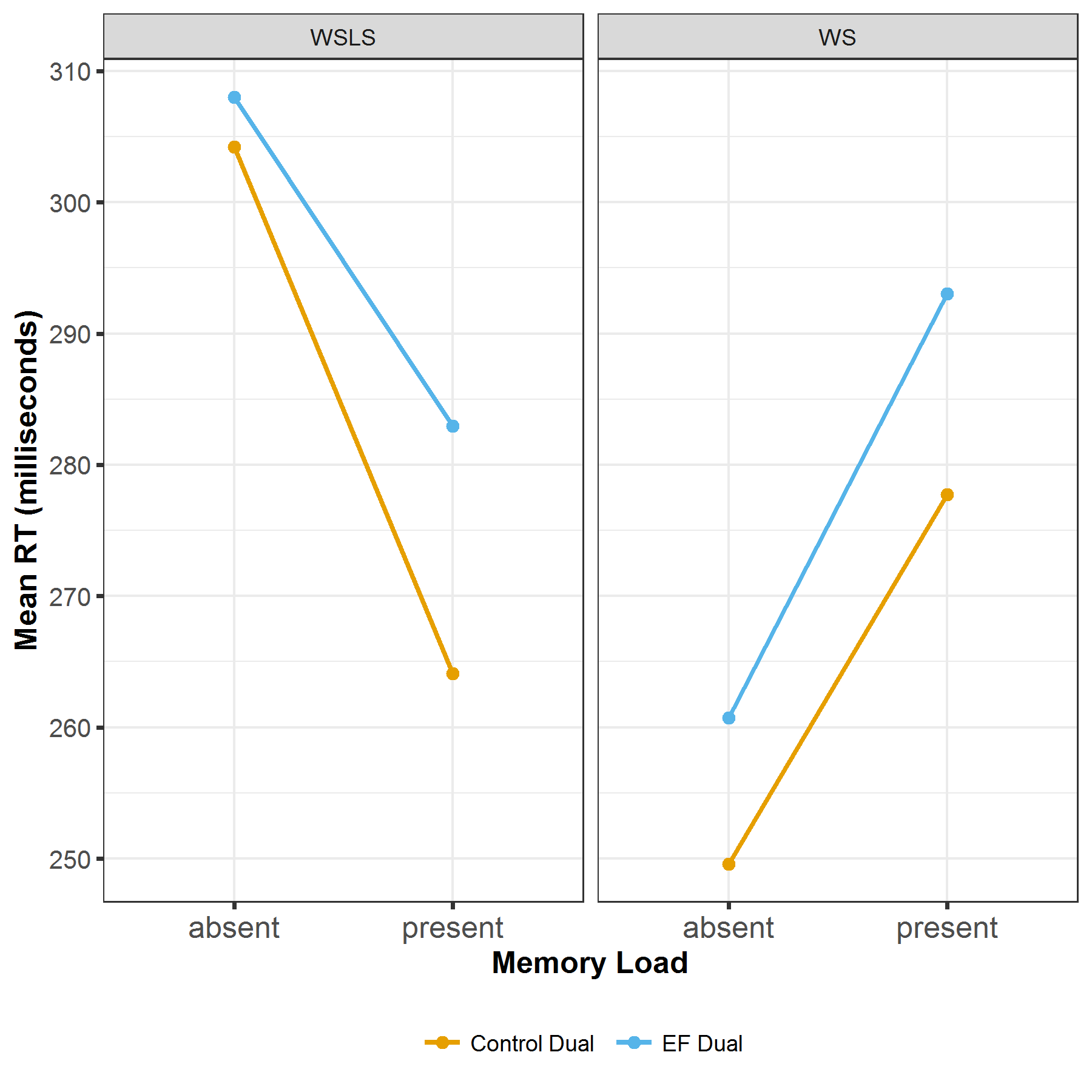


Figure S5: Change in RT with memory load, split by block condition and group

Group and response number: (b=-130, SE=11.7, t(19247)=-11.2, p<.001):
At R1 both groups had similar RTs (b=-15.6, SE=18.2, z=-0.856, p=.394) but at R2 the WS group had significantly faster RT than the WSLS group (b=46.7, SE=18.2, z=2.56, p=.012), indicating a greater speed increase between responses in the WS group compared to the WSLS group (see figure S6).


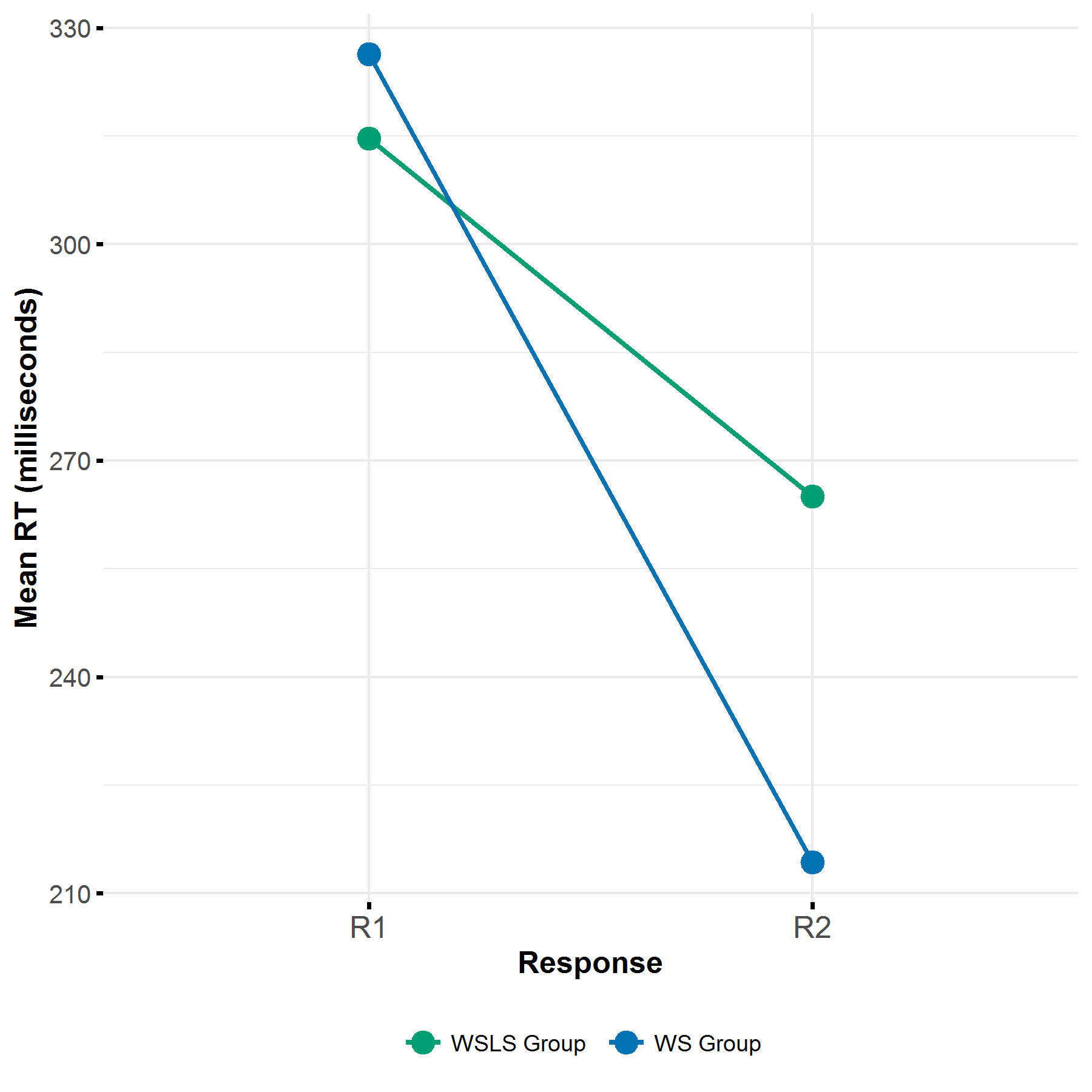


Figure S6: Speed increase between R1 and R2 for each strategy group

**Memory load and response number:** (b=-88.0, SE=11.5, t(19247)=-7.64, p<.001):
There was a significant decrease in RT between R1 and R2 overall both with and without a memory load (p<.001 for both memory conditions), but the decrease was larger when there was a memory load present (see figure S7).


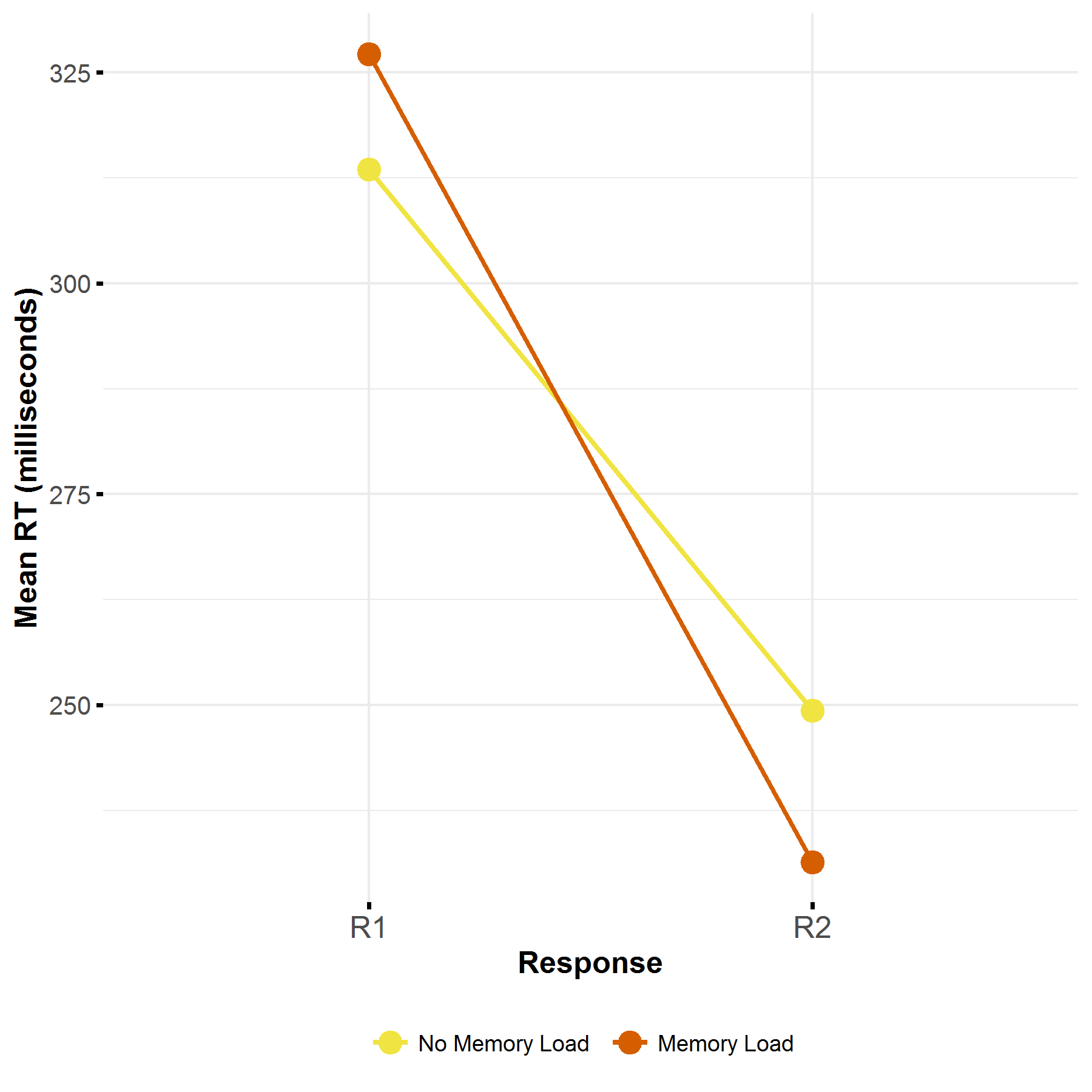


Figure S7: Reaction time decrease comparison between memory load and no memory load conditions.

Group, block condition, memory load and response number:


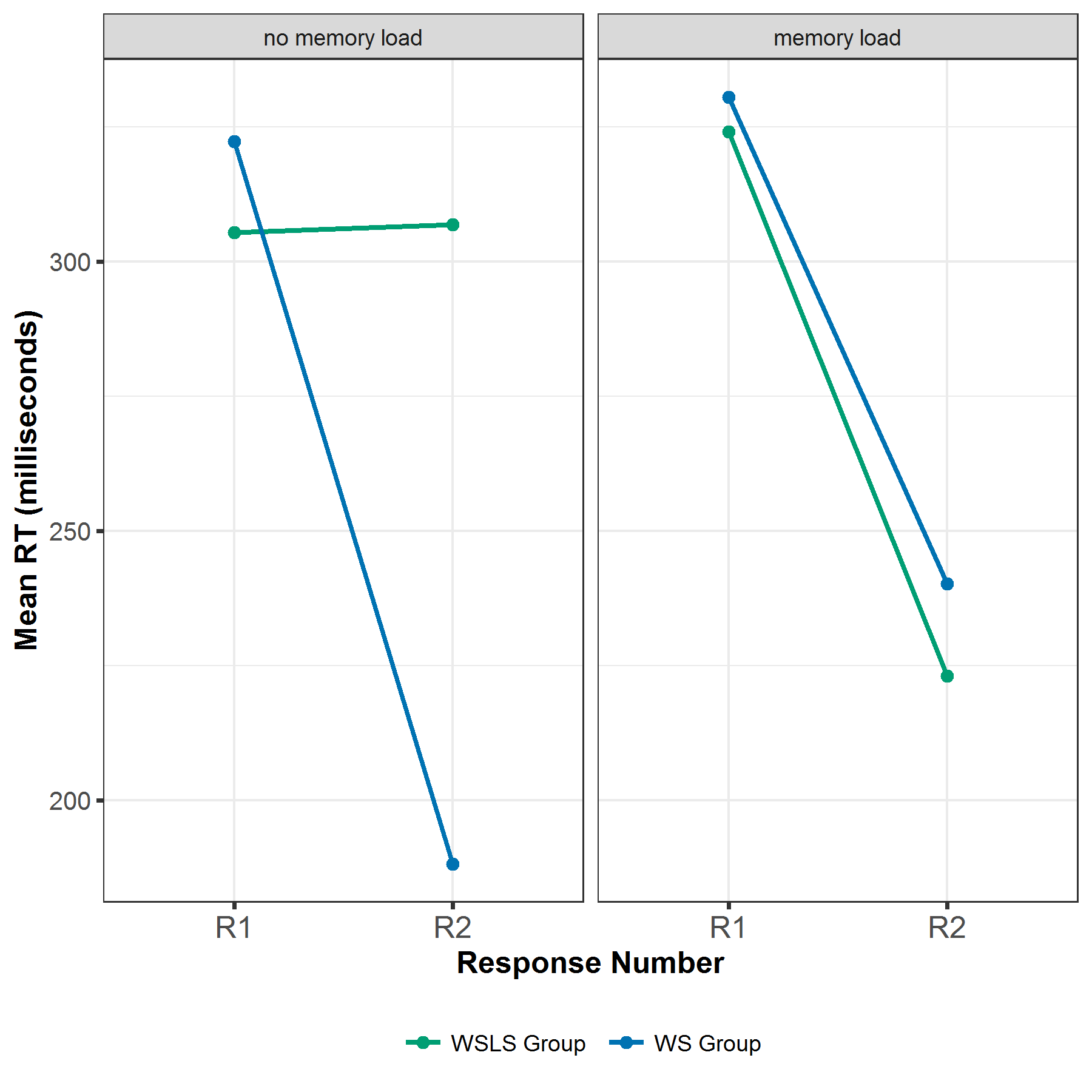


Figure S8: Speed increase from R1 to R2, split by memory load and strategy group.

## Analysis of Dual Task Data: E2

Participants all completed a different number of trials of the dual task, as their total trial number depended on the speed at which they completed the search and metacognition tasks, and which task they were doing. Analysis of the dual task performance was therefore capped at 84 trials, as this was the minimum number of trials completed by all participants in either block in both tasks (range metacognition EF block: 84-126; metacognition control block: 107-129; search EF block: 97-132; search control block: 123-139. The numbers of trials in the EF blocks are lower due to approximately one in six trials being a switch cue and not being counted in the analysis).

Participant accuracy was at or close to ceiling in all conditions (see figure S4); accuracy range 86.5%-95.7%.

Success on each trial of the audio task was analysed using a binomial linear mixed effects model with fixed effects of block condition, main task and their interactions, and scaled item number. Participant ID was included as a random effect. This model was significantly better than the null model (χ2(4)=850, p<.001). Accuracy was significantly lower in the EF block (b=-1.21, SE=0.057, z=-21.3, p<.001) but significantly higher during the search task compared to the metacognition task (b=0.219, SE=0.069, z=3.16, p=.002). Accuracy got lower as trial number increased, (b=-0.421, SE=0.071, z=-5.91, p<.001). There was also a significant interaction between main task and block condition (b=0.249, SE=0.085, z=2.91, p=.004), with a greater accuracy difference between control and EF blocks for trials completed alongside the metacognition task than the search task.
