# Supplementary material for: Limited evidence for executive function load impairing selective copying in a win-stay lose-shift task
Source: PLoS One. 2021 Mar 4;16(3):e0247183. doi: 10.1371/journal.pone.0247183 (PMC7932141; doi:10.1371/journal.pone.0247183)
Supplement: S2 File — (DOCX) [file pone.0247183.s018.docx]

Supplementary Information: Pilot Study

The aim of this pilot was to establish which dual-task method (if any) caused the greatest interference with reaction times (RTs) in the binary search task, with the intention of using this task combination with a larger sample size. Comparisons are drawn between change in RTs relative to no dual task, and to a control dual-task that is equally physically demanding but with no EF requirement. This is to ensure that dual-task interference is only attributed to competing EF demands where appropriate. The task(s) that elicited a significantly slower reaction time when under EF load than when in control conditions would then be considered for further testing.

### Methods

**Participants:**

Participants were recruited at the University of Stirling and took part in partial fulfilment of a course requirement to participate in research studies. Two participants chose to receive cash remuneration instead and were paid at a rate of £5/hour. Sixty three participants took part (eight male, mean age: 20, range: 17-45). Of these, four were excluded due to computer errors meaning they could not finish the task. A further 17 participants completed the training phase of the experiment but did not score above the inclusion threshold for the full testing phase and so left the experiment after they had completed training (see *procedure* for details). In total 42 participants (seven male, mean age:20.1, range: 17-40) completed the full procedure. Only data from this group of 42 has been included in the below analysis. All participants had normal or corrected to normal vision and hearing. Participants all gave written consent to take part and were aware that they were free to withdraw from the study at any time. Ethical approval for the study was given by the University of Stirling General University Ethics Panel (reference GUEP 111).

**Apparatus:**

Participants were tested using a desktop computer running Windows 8 with a standard mouse, a Black Box Toolkit 4 button response pad button box and Sony MDR-Pro over-ear headphones.

**Task Design:**

All tasks were written and run in Psychopy version 1.84.2. All participants completed the same main task, the binary search task detailed below. The binary search task was completed either on its own (baseline condition), or with one of five different dual task methods (four audio, one visual), designed with the intention to place demands on executive function. Individual dual tasks were between-subjects variables, with each participant only completing one dual-task. Each task designed to impair executive function (EF-task) was paired with a matched task that required the same motor response to be performed but with a substantially reduced executive function load (control task). This was a within-subjects variable, with each participant completing an EF-task and a control task, counterbalanced for order. Participants in the baseline condition completed only the search task. The baseline condition was not included as an additional within-participants variable in order to avoid fatigue and/or practise effects from participants having to complete a large number of trials of the same main task.

Brief descriptions of each dual task are given in table P1, with detailed descriptions given in the supplementary information.

Table P1: Brief descriptions of each EF dual task, and the matched control dual task. Task 1 is based on the dual-task used by Qureshi, Apperly, & Samson (2010); tasks 2-4 are based on the dual-tasks used by Bull, Phillips and Conway (2008), and task 5 is based on the task used by Coutinho et al. (2015) which was argued to inhibit metacognitive responding in humans and monkeys.

| **Dual Task** | **EF Task** | **Control Task** |
| --- | --- | --- |
| 1: *Incongruence* | Participants listened to a series of auditory tones (either 1 or 2) through headphones and responded with the opposite number (i.e. either 2 or 1) of mouse clicks. | Identical to the EF task, but participants responded with the same number of clicks as tones. |
| 2: *Inhibition (withholding)* | Participants listened to a series of auditory tones through headphones and responded to each tone with a mouse click, except under certain specified conditions when they were required to withhold their response. | Identical to the EF task, but participants responded to every tone with a mouse click with no conditions requiring withholding a response. |
| 3: *Switching* | Participants listened to a series of auditory tones through headphones and responded to each tone with a pre-specified mouse response. After an auditory cue they were required to switch to a different pre-specified mouse response. | Identical to the EF task, but participants would continue with the same mouse response for the duration of the task. |
| 4: *Updating* | Participants listened to a series of auditory tones, in sets of either 1 or 2 tones, through headphones. They responded by clicking the mouse once more than the number of tones heard in the previous set. | Identical to the EF task, but participants responded to the number of tones heard in the current block. |
| *5: Working Memory* | Participants were presented with two numbers on screen, one of which was clearly much larger in font size than the other, and one of which was larger in numerical value than the other. Participants were shown the two numbers briefly, and asked to remember them until cued to respond with either the number that is large in font size, small in font size, large in value, or small in value. | The same format as the EF task, but participants were just shown fixation crosses rather than asked to remember numbers. Instead of being asked to recall values participants were asked a very simple arithmetic question. |

Binary Search Task

This was a simple two alternative forced-choice (2AFC) binary search task, intended to assess how quickly participants could use vicariously presented information to make decisions. Participants were presented with an information trial which showed two shape stimuli on screen. After a brief pause the task revealed the value (successful – a fish was shown -or unsuccessful – a shark was shown) of one of the two stimuli. Participants were then required to make one selection from the two stimuli presented again (see figure 1, and the supplementary information). Participants were always instructed to find the fish and avoid the shark, and were awarded one point on trials in which they correctly selected the stimuli that displayed the fish. Reward location was fixed, so a WSLS strategy was always successful. The task therefore models the most basic requirement for cumulative cultural evolution discussed in the main text (section 1.1).

**Procedure:**

Participants were tested individually, although the majority of participants took part in the same room and at the same time as another participant. When two participants took part at once both participants were verbally instructed by the experimenter that they were acting entirely independently and were not competing with one another. Participants were facing away from each other so could not see each other’s screens. They were either both taking part in silent tasks or were both wearing headphones so audio distraction from the other participant was minimal. Both participants began the task at the same time and if one participant finished in a quicker time than the other they were escorted quietly out of the testing room to ensure the remaining participant was not disturbed.

Before beginning trials participants received detailed on-screen task instructions which included examples of the audio sounds they would be exposed to and the visual stimuli they were looking for. Participants then completed 4 practise trials of their audio dual-task on its own, followed by 2 practise trials of the binary search task. Participants in the *baseline* condition completed 2 trials of practise of the binary search task only, and participants in the *working memory* dual task condition completed 2 trials of the binary search task and dual task together. After this brief practise, participants were then required to complete 16 trials of pre-test training in each condition (EF load and control), with the order of conditions counterbalanced across participants (*baseline* participants did 2 blocks of 16 trials of the same task) in full dual-task conditions. For the audio dual tasks the number of trials was determined by the time taken to complete the binary search task. For the working memory dual task participants completed 16 trials per block.

[figure P1 approximately here]

Figure P1:
**LEFT:** Example trial of the binary search task being completed with an audio dual task. This is an example of an unsuccessful information trial (shark is displayed). **RIGHT:** Example trial of the binary search task being completed with the working memory dual task. This is an example of a successful information trial (fish is displayed). In both cases the test trial is successful (stimulus showing a fish is selected).

To ensure full focus was given to both tasks an inclusion criterion of 75% accuracy was set for both tasks, with a larger participation reward available to participants that completed the full study. Participants that scored below 75% accuracy in the training round (averaged over both blocks) in one or both tasks left the study at this point and received a smaller reward (a reduced participation fee, or fewer research participation tokens, relative to participants completing the full study). Participants were made explicitly aware of this inclusion criterion when signing up to take part in the study. They were also reminded of this again when giving consent to take part, and then once more as part of the written instructions for the study.

Participants who passed the training round then completed a further 48 trials of testing in each condition in full dual-task conditions (*baseline* participants did 2 blocks of 48 trials of the same task). For the audio dual tasks the number of trials was determined by the time taken to complete the binary search task. For the *working memory* dual task participants completed 48 trials per block.

### Results

The aim of this study was to select a task or tasks that proved effective in restricting participants’ ability to effectively use social information to make decisions. The results summarised here are therefore fairly brief, and focus only on whether significant differences were found between the response times in the binary search task for the baseline condition and each dual task, and whether there were significant differences in response times between the EF and Control dual task.

The study aim was to recruit 8 participants into each dual task condition, and 8 participants into the baseline. This was achieved for all tasks apart from *updating*. This was due to large numbers of participants in the *updating* condition scoring lower than the inclusion criterion during training (12 out of 14 tested without software issues), meaning data collection was halted for this condition. The results summarised below have therefore been conducted with the *updating* condition omitted.

The variable of interest was the reaction time of all responses given in the main task. Response time was used rather than accuracy as accuracy was predicted to be at ceiling levels in the task. Figure 2 shows the mean RT in the binary search task for each dual-task type. Accuracy in this task was approximately at ceiling across all task conditions (accuracy range: 96.4%-98.9%), so is not analysed here.

*Outliers*

Overall 186 outliers were removed from the data for very long or very quick reaction times. Each outlier represents a single trial. A relatively broad inclusion criterion (3 x Median Absolute Deviation (MAD) from the mean; Leys, Ley, Klein, Bernard, & Licata, 2013) was used to ensure genuinely long reaction times caused by dual task interference, which were the expected outputs of the study, were not artificially trimmed. Outliers were removed per participant, per task type in line with recommendations from Ratcliff (1993) regarding outlier removal for reaction time datasets with high inter-participant variation. 182 outliers were removed from the upper end of the response distribution and 4 were removed from the bottom end. This represents 4.7% of the total data.

*Difference from baseline*

Each task’s difference from the baseline task was calculated using a linear mixed effects model for each task with one fixed effect of block condition (baseline, control or executive function block) and one random effect of participant ID. *P*-values were estimated from the resultant t-statistics with degrees of freedom being the number of observations minus the number of fixed parameters in the model (Baayen, Davidson, & Bates, 2008). For the *incongruence* and *withholding* tasks the model was not significantly better than a null model (incongruence: χ2=0.288, p=.866; withholding: χ2=5.18, p=.075) and the tasks showed no significant differences from the baseline condition in either block. Both the *switching* and *working memory* task models were significantly better than the null model (switching: χ2=43.6, p<0.001; working memory: χ2=21.2, p<.001) and both tasks showed significantly longer reaction times in each block compared to the baseline task. The results of each model are presented in table P2.

Table P2: Difference between average reaction time in milliseconds of each task from the baseline condition (details of models given in brackets)

| **Task** | **Control difference from baseline (ms)** | **EF difference from baseline (MS)** |
| --- | --- | --- |
| **Incongruence** | 9.05 (SE=27.0, t(1467)=0.335, p=.738) | 5.772 (SE=27.0, t(1467)=0.214, p=.831) |
| **Withholding** | 8.88 (SE=25.2, t(1473)=0.352, p=.25) | 24.8 (SE=25.3, t(1473)=0.980, p=.327) |
| **Switching** | 22.28 (SE=24.7, t(1453)=0.901, p<.001) | 80.8 (SE=24.7, t(1453)=3.27, p<.001) |
| **Working Memory** | 45.7 (SE=31.5, t(1490)=1.45, p<.001) | 74.9 (SE=31.5, t(1490)=2.38, p<.001) |

*Difference between control task and EF task*

The difference between the control and executive function blocks of each task were calculated using a linear mixed effects model for each task with one fixed effect of block condition (control or executive function block) and one random effect of participant ID. For the *incongruence* task the model was not significantly better than a null model (χ2=0.179, p=.672) and the task showed no significant differences between the executive function and control blocks. The *withholding, switching* and *working memory* task models were all significantly better than the null model (withholding: χ2=4.10, p=.043; switching: χ2=26.6, p<.001; working memory: χ2=16.3, p<.001) and all three tasks showed significantly longer reaction times in the executive function block compared to the control block. The results of each model are presented in table P3.

Table P3: Mean RT in ms to 3.s.f (SD in brackets) of each task block. Difference column shows results of linear mixed effects models testing if blocks were significantly different to each other.

| **Task** | **Control** | **EF** | **Difference** |
| --- | --- | --- | --- |
| **Incongruence** | 506 (113) | 502 (114) | b=-3.29, SE=7.80, t(721)=-0.422, p=.673 |
| **Withholding** | 505 (112) | 521 (111) | b=15.9, SE=7.83, t(727)=2.03, p=.043 |
| **Switching** | 518 (125) | 577 (177) | b=58.6, SE=11.3, t(708)=5.20, p<.001 |
| **Working Memory** | 542 (107) | 572 (124) | b=29.2, SE=7.18, t(744)=4.06, p<.001 |

[figure P2 approximately here]

Figure P2: RT for each task type split by dual-task block. Red dots indicate mean for each block. † indicates block is significantly different to the baseline condition. Brackets indicate significance of difference between control and EF block within a task.

### Discussion

The results presented above suggest the *switching* or *working memory* tasks could both be used as viable dual-tasks to experimentally restrict participant ability to make rapid decisions using social information, as they both produced a significant difference between the control and executive function blocks, and the executive function blocks were significantly different from the baseline condition. This slowing down of responses in these conditions indicates that these executive functions are involved in making inferences with vicarious information (although it should be noted that this effect may not be peculiar to use of social or vicarious information and might also apply to learning from feedback on one’s own previous choices). Both tasks also showed significant differences between the control block and the baseline task. This is to be expected, as doing anything at the same time at the main task is likely to increase reaction times, and highlights the importance of including a control dual task as well as a baseline and a test condition. Although there was also a significant difference between control and EF blocks in the *withholding* condition, as neither block of this task presented a difference from the baseline condition this would not be an appropriate dual-task to continue to use within this testing paradigm. Previous literature implicates *updating* and *inhibition* most strongly in metacognition tasks (Roebers 2017). The different result found here may highlight the importance of empirical testing to establish sound methods for data collection. However, this could also indicate that although executive functions are playing a formative role in the learning decisions required for CCE to occur, metacognition may not be implicated.

The *incongruence* task showed no significant RT difference, when completed under control and executive-function tasks, suggesting the executive function load these tasks placed on participants was not large enough to impair performance on the binary search task, or that the type of EF required to complete the *incongruence* task is not required to make WSLS decisions.

As the data from the *updating* task are so limited, a definitive conclusion about this task type cannot be drawn. It is clear from this specific set of results that the task in the way it was designed for this study was too challenging for the majority of participants. However, that does not mean tasks utilising the updating component of executive function would never be useful as dual-tasks.

While *switching* and *working memory* both showed significant results, only 1 dual-task method at a time was sought to take forward for further testing. This was to ensure all participants will have their executive functions impaired in the same way, to ensure comparable data across the entire task. For the subsequent studies presented here just the *switching* task is used. This task was chosen as it was faster to complete than the *working memory* task, allowing for more trials to be completed within the same testing period. The *working memory* task may be used in future testing.
