# Supplementary material for: Limited evidence for executive function load impairing selective copying in a win-stay lose-shift task
Source: PLoS One. 2021 Mar 4;16(3):e0247183. doi: 10.1371/journal.pone.0247183 (PMC7932141; doi:10.1371/journal.pone.0247183)
Supplement: Attachment — Submitted filename: PLOS rebuttal letter.docx [file pone.0247183.s020.docx]

Psychology
Faculty of Natural Sciences
University of Stirling
Stirling
FK9 4LA
UK

Email: julietdunstone@gmail.com
[c.a.caldwell@stir.ac.uk](mailto:c.a.caldwell@stir.ac.uk)

Tuesday 20^th^ December 2020

Dear Professor Le Pelley,

Thank you for these comments from yourself and the two reviewers. We found these comments to be constructive and extremely helpful in improving the clarity of the manuscript.

I have submitted a revised version of the manuscript with changes written in red text, and I have detailed below how I have addressed the points raised. I have not used track-changes in the manuscript as a large proportion of the suggested edits were to move sections into supplementary information and using the tracked changes format for removing large sections makes reading the revised manuscript very challenging. I hope that the format I have used is acceptable.

Thank you for giving us the opportunity to make these revisions,

Juliet Dunstone

**Editors points:**

I will not reiterate all of the points made by the reviewers since the reviews they have provided are clear and well-argued. It will be important for you to address all of these points in any revision. I will merely highlight some of the key issues here, and add thoughts from my own reading of the paper.

1.  Both reviewers note that the manuscript feels over-long, and I agree with them: as it stands there is a danger of the relatively straightforward message of the paper becoming lost in a mass of methodological detail and statistical analysis. I would therefore encourage you to streamline the article wherever possible. For example, both reviewers suggest moving the details of the pilot experiment to supplementary materials (Reviewer 1 suggests also moving Experiment 1 - I will leave that decision up to you). Likewise it felt like the results sections could be condensed: at the moment it sometimes feels a little like every possible contrast of every dependent variable is being reported, and I found myself getting lost in a sea of statistics. Again I encourage you to strip back the reported analyses to what you consider the most critical aspects that address the hypotheses that you have developed in the introduction (moving less focal analyses to supplementary materials as appropriate). As just one example, I suggest omitting the analyses of performance on the secondary task from the main text.

The pilot task has now been moved to supplementary information. Experiment 1 has not been moved into the SI as this is the only part of the task which compares two different types of single-action copying. Both sets of analysis of the concurrent dual-task have been moved into the SI, as have two of the interactions from experiment 2.

2.  I also wondered if the introduction could be shortened. The paper is currently themed around the idea of cumulative cultural evolution, but ultimately comes down to a question about cognitive processes that need not be tied to CCE – much as you note at lines 179-182. On that basis, it felt like some of the background on CCE might perhaps be superfluous (e.g., lines 95-103; lines 113-122). Also, on the topic of length: (1) the abstract should be 300 words max, and (2) you could omit Table 4, given that these data are also shown in Figure 3. Likewise the table shown at line 722 (which does not have a table number).

I have chosen to leave the content about CCE in the introduction, as it was the theories from Heyes and others regarding CCE that led us to use a dual-task method in the first place, so removing these aspects would not accurately portray the motivations for the study. As reviewer 2 points out, some of the findings are also relevant within the context of human CCE.

I have also chosen to leave the tables as well as figures, as I believe this makes the article more accessible for anyone reading with any visual impairment or using screen reading technology.

The abstract has been re-written to be under 300 words and all table numbers have been updated.

3.  Reviewer 2 notes that “the task simulates making inferences with social information, but is not a social learning task in itself”. I agree, and found the title a little misleading in this regard: it led me to expect that the study would be investigating (specifically) social learning strategies.

 This point has been clarified in line with reviewer 2’s comments, and the title has been updated

4.  I don’t think it’s appropriate to describe the 2AFC task as being a “search task”. Visual search has a specific meaning in the cognitive literature, Wikipedia gets it about right: “Visual search is a type of perceptual task… that typically involves an active scan of the visual environment for a particular object or feature (the target) among other objects or features”, e.g., searching for a red item among green items. Your task is more about choosing an option than finding an option; it would be better described as a “binary response” task or a “binary choice” task than a search task.

This has been updated throughout to be a choice task rather than a search task

5.  Am I right in thinking that the WSLS condition in Experiment 2 is the only condition out of both experiments in which participants could not always deduce the correct answer(s)? As I understand it, if they are presented with one correct answer and one incorrect answer (as in the example in Fig 5), they would have to guess (with 50% success rate) one of the correct locations. If so, please could that be clarified.

 This has been clarified now

6.  Line 735: “The EMCC would predict that the dual-task would have more of an impact in the WSLS group, so would therefore predict longer RTs in the EF block of the selective group, when compared to the control and to the WS group. Longer RTs would also be predicted in the EF block compared to the control block, and when under an additional working memory load. These predictions are supported by the significant interaction between group, block condition and memory load”. The last sentence here is potentially misleading, since it implicitly attributes the source of the three-way interaction to the set of predictions that has just been described, and ignores the fact that there are unpredicted aspects of the findings that are also contributing to this interaction. For example, under no-memory-load conditions, the EF dual task seems to create an impairment for the WS task but not the WSLS task. It’s hard to see how this follows from the EMCC.

Some clarification has been added to indicate that higher dual-task impact on the WS group is not predicted by EMCC, but with a note that overall RT changes are affected by the difference between R1 and R2. This then directs the reader to the discussion about ‘planning to remember’.

7.  A few more minor / specific points:

-  Line 446: “Participants who passed the training round then completed a further 72 trials of testing in each condition in full dual-task conditions”. When I first read this I assumed that “each condition” referred to learning-strategy conditions which was to be manipulated within-subjects, but having read on I guess this must instead have referred to EF and control versions of the dual task. Is that correct, and if so please could it be clarified in the text? This has been clarified in-text

-  Please could it be clarified in the text that when you are talking about the “audio task”, you are talking about the switching task that’s being used as a secondary task? This has now been updated wherever it could be unclear

-  Line 495 (and other places): “A post-hoc Bonferroni-Holm correction of all pairwise comparisons of group membership indicated” should be something like “Post-hoc pairwise comparisons of group membership using Bonferroni-Holm correction indicated…” This has been corrected

-  The Figure 3 caption is too ready to accept the null hypothesis. This should be changed to something more like: “There was no SIGNIFICANT difference overall between the LS and WS groups. The difference between the control and EF block condition was NOT SIGNIFICANTLY DIFFERENT for each strategy group.” This has been updated

-  We probably don’t need all the detail on what happened during the between-block breaks (lines 644-650). It’s enough to say that participants took a 5-min break between phases. This has been removed

**Journal requirements:**

1. Please ensure that your manuscript meets PLOS ONE's style requirements, including those for file naming. The PLOS ONE style templates can be found at

https://journals.plos.org/plosone/s/file?id=wjVg/PLOSOne_formatting_sample_main_body.pdf and

https://journals.plos.org/plosone/s/file?id=ba62/PLOSOne_formatting_sample_title_authors_affiliations.pdf

2.  1) Please consider changing the title so as to meet our title format requirement (https://journals.plos.org/plosone/s/submission-guidelines). In particular, the title should be "Specific, descriptive, concise, and comprehensible to readers outside the field" and in this case it is not informative and specific about your study's scope and methodology. A new title has been given to the manuscript.

2) You indicated that you had ethical approval for your study. In your Methods section, please ensure you have also stated whether you obtained consent from parents or guardians of the minors included in the study or whether the research ethics committee or IRB specifically waived the need for their consent.- A clarification has been added within text to say 16-18 year olds are considered adult participants according to British Psychology Society guidelines

3) In your manuscript, please include the information removed for blind review since PLOS ONE's peer-review process is not double blinded (<https://journals.plos.org/plosone/s/editorial-and-peer-review-process>). The blinding has now been removed.

3. Our internal editors have looked over your manuscript and determined that it is within the scope of our Cognitive Developmental Psychology Call for Papers. The Collection will encompass a diverse range of research articles in developmental psychology, including early cognitive development, language development, atypical development, cognitive processing across the lifespan, among others, with an emphasis on transparent and reproducible reporting practices.  Additional information can be found on our announcement page: https://collections.plos.org/s/cognitive-psychology.  If you would like your manuscript to be considered for this collection, please let us know in your cover letter and we will ensure that your paper is treated as if you were responding to this call. Please note that being considered for the Collection does not require an additional peer review beyond the journal’s standard process and will not delay the publication of your manuscript if it is accepted by PLOS ONE. If you would prefer to remove your manuscript from collection consideration, please specify this in the cover letter.

We do not have a strong preference about whether or not to be included in this collection, so if possible are happy to leave this decision up to the editor as to whether they deem it a suitable fit for the collection.

4. Please amend the manuscript submission data (via Edit Submission) to include author: Juliet Dunstone. Juliet Dunstone and Juliet Robertstone are the same person (names pre- and after marriage). The submitted name should be Juliet Dunstone as this is the name linked to the first author’s orcid ID and the same name given on previous publications. Submitting under the name Juliet Robertstone was an error in setting up the online account with PLOS which has now been rectified.

5. Please amend your authorship list in your manuscript file to include author: See above comment.

[Note: HTML markup is below. Please do not edit.]

Reviewers' comments:

Reviewer's Responses to Questions

**Comments to the Author**

1. Is the manuscript technically sound, and do the data support the conclusions?

The manuscript must describe a technically sound piece of scientific research with data that supports the conclusions. Experiments must have been conducted rigorously, with appropriate controls, replication, and sample sizes. The conclusions must be drawn appropriately based on the data presented.

Reviewer #1: Yes

Reviewer #2: Yes

2. Has the statistical analysis been performed appropriately and rigorously?

Reviewer #1: Yes

Reviewer #2: Yes

3. Have the authors made all data underlying the findings in their manuscript fully available?

The [PLOS Data policy](http://www.plosone.org/static/policies.action#sharing) requires authors to make all data underlying the findings described in their manuscript fully available without restriction, with rare exception (please refer to the Data Availability Statement in the manuscript PDF file). The data should be provided as part of the manuscript or its supporting information, or deposited to a public repository. For example, in addition to summary statistics, the data points behind means, medians and variance measures should be available. If there are restrictions on publicly sharing data—e.g. participant privacy or use of data from a third party—those must be specified.

Reviewer #1: Yes

Reviewer #2: Yes

4. Is the manuscript presented in an intelligible fashion and written in standard English?

PLOS ONE does not copyedit accepted manuscripts, so the language in submitted articles must be clear, correct, and unambiguous. Any typographical or grammatical errors should be corrected at revision, so please note any specific errors here.

Reviewer #1: Yes

Reviewer #2: Yes

5. Review Comments to the Author

Please use the space provided to explain your answers to the questions above. You may also include additional comments for the author, including concerns about dual publication, research ethics, or publication ethics. (Please upload your review as an attachment if it exceeds 20,000 characters)

Reviewer #1: The paper presents a series of cleverly and intricately designed and analysed studies to address the extent to which executive functions (as a proxy for explicit metacognition) are involved in flexible use of social learning strategies. The paper is very lengthy and may be better presented as two good and necessary preliminary studies (the current pilot study and study 1 which is not super representative of the cumulative culture context and had an issue with ceiling effects) with much of the text in supplementary information. This would allow focus on the more ecologically valid study 2

The pilot has been moved to supplementary information, please see above comment to editor for details.

which also incorporated valuable elements of measuring memory load and, crucially –given the focus of the introduction- metacognition. Given this latter focus the authors are careful not to over-claim from their results and the conclusion, abstract and title reflect this. Given the aim of the paper it would be interesting to hear why a more obviously metacognitive proxy for a flexible social learning strategy underlying cumulative culture was not used, rather than the WSLS paradigm. For example, where an individual has to judge the quality of their own knowledge/social information before determining whether to adopt new social information/stick with their own knowledge (and or innovate).

Overall the wider project that this study was a part of specifically looked at very simple methods of eliciting ratcheting across multiple study populations, so using the ‘WSLS’ style task fit with the overall scope of the research. The study presented here aimed to replicate similar data that was collected with young children – this point has now been added in to the manuscript. A more complex task may have involved metacognition more directly, but this would not have been able to look at the specific ratcheting potential element of each decision to be made, and finding an overtly metacognitive task to use as a dual-task that wasn’t so distracting that it made completing the main task impossible was a challenge – this is why the proxy task was adopted. Clarification regarding this decision has also been added into the manuscript.

There are many clarity issues that are easily resolved with consistent use of terminology (see below comments).

L47 – it would be helpful to explain what distinguishes the first from the second study (what is different if anything?).
L50 – it is not until this line of the abstract that any mention of measuring metacognitive ability is made. This is odd as the abstract introduces the point of the study as being to do with testing the hypothesis about human metacognitive ability and our cultural abilities. [perhaps the flexible strategy condition equates to increased metacognition but this is not stated].
L56 – use past tense. (‘…. could have been ….’)
L57 – what does “such contexts” refer to? This is not clear.

All of the above points refer to the abstract, which has now been largely reworded in order to reduce the length. I aimed to reword the abstract to ensure these clarity issues were resolved as well.

L84 – doesn’t make sense ‘… away from unhelpful…’

Clarification has been added to this sentence

L102-3 – a caveat should be added here that obviously being able to ‘verbalise’ a strategy is not the criteria that distinguishes humans from nonhumans regarding metacognition as non-human animals cannot speak. Also, there has been a fair amount of research into metacognition in nonhumans that really ought to be mentioned and incorporated into your argument. I realise that you are not attempting a comparative study or attempting to address the ‘human unique’ aspect of the theory (it would be good to state this explicitly early on) but the intro discussion of the theory seems incomplete as is.

The manuscript has been updated with this caveat, and with a reference to a paper that contains a summary of non-human metacognition studies and how they relate to the question presented in the current study.

L151 – it is a bit presumptious to assume that absolutely all nonhuman SLSs have to be non-explicit. We haven’t really tested this properly in nonhumans. Use of words like ‘likely differentiates’ or ‘may differentiate’ would be more appropriate. This has been edited to say ‘likely differentiates’

Table 1: working memory task – clarify what the ‘test trial’ refers to as it is not mentioned in this way in the rest of the table (consistency in terms) – The description of the control wm task in the pilot study has been updated to improve the clarity.

L318 & L472 – clarify why removal of outliers important for these data. This has been included

L368 – without comparing the influence of concurrent EF tasks to social and non-social WSLS tasks, can you actually say that working memory and switching are specifically involved in the use of social info.? This has been updated to acknowledge that the information source is vicarious rather than truly social, with an additional acknowledgement that we cannot confirm whether behaviour would be different if the observed information was self-generated or truly social.

L450 – check for consistency of condition terms used – strategic copying is used here but elsewhere flexible, selective etc. Terms have been updated to be consistent throughout

L452 – replace ‘showing’ with ‘observing’ so the condition is from the point of view of the participant This has been updated

L454 – clarify what ‘rewarded’ and ‘unrewarded’ means – is it that they observed the location of the desired fish or undesired shark? Is it the same as the ‘successful or unsuccessful information trials’ on L507 or ‘informational trial type’ in Fig 4? The manuscript has been updated to refer to successful/unsuccessful trials instead of rewarded / unrewarded trials

L463 – complete relative statements “slower” than what? This has been updated to clarify it means slower than the control block

L491 – what is a ‘strategy group’? I’m guessing the conditions but consistency in terms will help the reader. Similarly on L497 we get ‘response type’ conditions and on L596 there is mention of ‘repeated copying condition’. This has been clarified from ‘strategy group’ and condition to just ‘group’, and it has been clarified what is meant by repeated copying condition

L578 – is the WSLS really the “only condition analogous to a social learning strategy”? or did you mean the only one analogous to a SLS that could support cumulative culture? WS or LS could both be described as content-dependent SLSs. This has been updated to say the only condition analogous to an explicit SLS. Further clarification on the differences we see between the WSLS and WS/LS conditions have also been added to the manuscript: “Employing a blanket always-copy or never-copy could be considered strategies if they were applied to variable input. However, in the context of this experiment they are not, as they are not being used conditionally: In the WS condition participants only observe wins, and in the LS condition participants only observe lose trials so the conditions themselves aren’t strategic.”

L595 – by ‘two different strategies’ do you mean ‘both WS and LS’? If so I think the latter is clearer. This has since been clarified

L620 - Can you clasrify that participants in study 2 were task naive and hadn’t taken part in the pilot or study 1. Yes, this is confirmed now

L680-681 – the explanation of points being ‘equal to the confidence rating given’ is not clear (procedurally or reasoning wise). Suggest move the explanation given from L800 (in the results) up to the Methods here. A more detailed explanation of the scoring system has now been included

L686 – ‘in conjunction’ is unclear, do you mean the metacognition trials were interspersed with the search trials and for both participants had to do the audio switching task concurrently? It has now been clarified at the start of the method section what the design was (metacognition and WSLS not interspersed, both tasks done with concurrent dual task), and the wording has been updated to say concurrently not ‘in conjunction’

L948 – the discussion/conclusion could do with returning to the argument of why explicit metacognition is thought to be important to cumulative culture and what your results have to say about this (with the appropriate caveats/cautions). Why was the conscious aspect of metacognition not addressed by asking participants whether they were aware of using a strategy and what it was?

Additions to discussion have been added in address the first point.

With regards to the second point: participants were verbally asked about their strategies, but few were forthcoming with responses. The response was almost universally along the lines of “I tried to find the fish as quickly as possible without getting distracted”. Indeed, very few participants were interested in engaging with debrief information or discussing their participation. This may have been due to the population sampled (undergraduate participants, who regularly take part in psychology studies as part of their course requirements). Thus I can provide no further information about this.

Reviewer #2: This is a thoroughly investigated and reported study on whether some social learning strategies are explicitly metacognitive, by requiring participants to complete an executive function (EF) task while simultaneously completing a learning task that requires a flexible learning strategy. the authors report a pilot study and two experiments. In experiment 1 the EF shadowing task had a comparable effect on both the flexible learning task and two control tasks. In experiment 2 the difficulty of the flexible learning task was increased by incorporating a memory component and increasing the number of stimuli to be learned about in each trial. In this case there was a clearer effect on the flexible learning task than on the control task. The results are important within the context of recent theories of human cumulative cultural evolution.

The rationale for the study is very clearly and concisely put forward in the introduction. However, although I can understand the authors' thoroughness in reporting their study, the overall report is long and rather bloated. Essentially this study asks a simple question - does an executive function task affect a learning task in which the participant has to respond flexibly more than a task in which they don't. There is a lot of methods and results material for the reader to get through to find out the answer to this, and my main recommendation is to cut some of the report to make the story easier for the reader to follow. To that end, I would recommend putting the pilot study into a supplement - only the most engaged reader will want to know how you came to choose the EF task you did.

The nature of the flexible learning task (win-stay lose-shift) is alluded to in numerous places in the introduction but is never explained in concrete terms. I struggled to understand what the participant actually did until I delved into the supplementary materials, so I think the WSLS task has to be much clearer front and centre.

minor points
line 113. could you be clearer when you say 'many focus on different aspects of cultural evolution that could be assessed differently'? What aspects, how could they be assessed differently? I assume you really want to say 'although there are multiple definitions of CCE, for our purposes Mesoudi and Thornton (2019) have provided to definitions we will use...' Agreed, I have streamlined this to make it clearer

line 369. Could you make it clear that the task simulates making inferences with social information, but is not a social learning task in itself. This is a fair point and has been clarified
line 397. The different conditions in this experiment are poorly explained in the introduction. Could you be clearer? More detail has been added to clarify the task conditions

line 582. It did occur to me that because I didn't fully understand what the participant did in the wsls task, perhaps there was as greater motor cost in this task than in the control tasks. Can the authors be sure that the EF task doesn't simply produce an interference effect on motor control? I can confirm that the motor cost of the tasks is the same in all conditions

line 721. I don't think the table provides any information that figure 7 doesn't also show. I would ditch the table and put figure 7 much earlier in the results section. Please see above comment to editor regarding figures and tables. I have moved the suggested location of figure 7 earlier on in the text.

line 731. Was baseline taken from R1 or R2? R1 – This is updated in the text
