# Supplementary material for: Limited evidence for executive function load impairing selective copying in a win-stay lose-shift task
Source: PLoS One. 2021 Mar 4;16(3):e0247183. doi: 10.1371/journal.pone.0247183 (PMC7932141; doi:10.1371/journal.pone.0247183)
Supplement: Attachment — Submitted filename: PLOS_second_revisions_rebuttal.docx [file pone.0247183.s021.docx]

Psychology
Faculty of Natural Sciences
University of Stirling
Stirling
FK9 4LA
UK
Email: [julietdunstone@gmail.com](mailto:julietdunstone@gmail.com)
Monday 25^th^ January 2021

Dear Professor Le Pelley,

Thank you for the further comments on my revised manuscript. I agree that these changes improve the clarity of the manuscript further.

I have submitted a revised version of the manuscript with the suggested changes made via track-changes and I have detailed below how I have addressed the points raised.

Thank you for giving us the opportunity to make these revisions,

Juliet Dunstone

1.  L211-212: some note should be made here of the dual task, which is a critical aspect of Experiment 1. E.g., perhaps something like “Experiment 1 therefore compared participant performance in a basic selection task when a selective strategy was required, compared to when it was not, under conditions in which participants performed a secondary task that was intended to place a high or low load on executive resources”.

This clarification has been added.

2.  L220: WS and LS should probably be defined in their ‘separate’ form (WSLS has previously been defined, but a reader may not make the connection without prompting).

A clarification explaining the individual meaning of WS and LS has been added.

3.  L263: It’s not clear to me what “vicariously presented information” means here. The information is presented directly to participants; they are not told information via a third party. I appreciate that an analogy is being made here to social learning, where a participant might see the outcome of somebody else’s behaviour. But regardless, in the current task the relevant outcome information is presented directly to participants.

“Vicarious” was used to make it clear that the information was presented without coming from a particular social source. However, I appreciate that this could be mis-interpreted so I have updated the wording accordingly to say ‘information they were presented with’.

4.  A general point regarding terminology relating to the audio task. In some places the conditions of this task are referred to as the “switching task” and “control task”, whereas in other places they are referred to as the “executive function / EF task” and “control task” (and notably, when the task is introduced in Methods [L276-288] there’s no mention that the switching task is also going to be called the EF task). Please can you make the terminology consistent throughout the paper, which will help the reader to keep track of which tasks you are talking about. On this topic, “switching task” seems like perhaps a better label than “EF task”, given that many of the tasks that you talk about in this study would place a load on executive function.

(Also on this point, in the abstract this talk is referred to as a “distractor task”, which is different yet again.)

One more (related) terminological issue that is somewhat pedantic. “Dual” means “consisting of two things”, so it doesn’t really make sense to call the audio task the “dual task”, because it is only a single thing. Instead it should probably be termed a “secondary” task. That is, the whole procedure is a dual-task design as it consists of two separate tasks, a primary task (e.g., WSLS) and a secondary task (e.g., audio switching).

This has been updated throughout to refer to the task as the secondary task when speaking about it generally, with the distinction between blocks being highlighted as switching / control rather than EF / control. This has also been updated in the figures.

5.  Reaction time is sometimes written out in full, and sometimes as “RT”. Given that your article already has a lot of abbreviations, I recommend writing it out in full each time.

This has been updated throughout to say “reaction time” in full, with the exception of figure axis labels in order to still keep the labels short and readable. Where the abbreviation has been used in an axis label it has been stated in full in the figure legend. In order to reduce the number of abbreviations the abbreviation ToM for theory of mind has also been changed to be written in full, as it was previously only used twice.

6.  L374-375: what was the correction for multiple comparisons that was used here? Bonferroni-Holm (like in the case mentioned at line 378)?

Yes, this has now been updated in text.

7.  L383, “the switching task had a negative impact on response times in each group”: please state what the negative impact is relative to (presumably the control task).

This has been clarified in the text.

8.  L408: This section is titled “Executive Function Dual Task” but talks about data from both the switching (EF) task and the control task (which is being considered as a non-EF task).

Both sections which briefly describe the results of the secondary task have now been re-titled ‘secondary task’.

9.  L412: Please write out “Supplementary Information” in full.

This has been corrected.

10.  L432-433: Please change to  “accuracy in the concurrent audio switching task also decreased (see Supplementary Information)”, since that is where the analysis of switching-task RTs is reported.

This has been added.

11.  L580-589: To avoid unnecessary repetition, this section could be replaced with “Overall 531 outliers were removed from the data for very long or very short reaction times, using the same procedure for outlier removal as in Experiment 1”.

This has been changed.

12.  L615-617: “The EMCC would predict that the dual-task would have more of an impact in the WSLS group, so would therefore predict longer RTs in the EF block of the selective group, when compared to the control and to the WS group”. The impact of the dual task is defined by the impairment in the switching (EF) condition relative to the control (non-EF) condition. So should the second part of this sentence instead be something like: “so would therefore predict a greater performance impairment in the EF block (relative to the control block) for the selective (WSLS) group than for the WS group”?

The wording of this prediction has been clarified according to your suggestion, to indicate the performance impairment rather than simply longer reaction times.

13.  L759:  “There was a significant decrease in concurrent audio switching task accuracy for both main task conditions, especially the metacognition task” – a decrease relative to what? Also, should there be a reference to Supplementary Information here?

This has been clarified to state the difference is between switching and control blocks and a reference to the supplementary information has been added.

14.  L779: “tasking switching” is a typo.

This has been corrected.

15.  L257 and L496: “Psychopy” should be written “PsychoPy”.

This has been corrected.
